# A prediction model for advanced colorectal neoplasia in an asymptomatic screening population

**DOI:** 10.1371/journal.pone.0181040

**Published:** 2017-08-25

**Authors:** Sung Noh Hong, Hee Jung Son, Sun Kyu Choi, Dong Kyung Chang, Young-Ho Kim, Sin-Ho Jung, Poong-Lyul Rhee

**Affiliations:** 1 Department of Medicine, Samsung Medical Center, Sungkyunkwan University School of Medicine, Seoul, Korea; 2 Center for Health Promotion, Samsung Medical Center, Seoul, South Korea; 3 Biostatistics and Bioinformatics Center, Samsung Cancer Research Institute, Samsung Medical Center, Seoul, Korea; 4 Department of Biostatistics and Bioinformatics, Duke University, Durham, North Carolina, United States of America; University Hospital Llandough, UNITED KINGDOM

## Abstract

**Background:**

An electronic medical record (EMR) database of a large unselected population who received screening colonoscopies may minimize sampling error and represent real-world estimates of risk for screening target lesions of advanced colorectal neoplasia (CRN). Our aim was to develop and validate a prediction model for assessing the probability of advanced CRN using a clinical data warehouse.

**Methods:**

A total of 49,450 screenees underwent their first colonoscopy as part of a health check-up from 2002 to 2012 at Samsung Medical Center, and the dataset was constructed by means of natural language processing from the computerized EMR system. The screenees were randomized into training and validation sets. The prediction model was developed using logistic regression. The model performance was validated and compared with existing models using area under receiver operating curve (AUC) analysis.

**Results:**

In the training set, age, gender, smoking duration, drinking frequency, and aspirin use were identified as independent predictors for advanced CRN (adjusted *P* < .01). The developed model had good discrimination (AUC = 0.726) and was internally validated (AUC = 0.713). The high-risk group had a 3.7-fold increased risk of advanced CRN compared to the low-risk group (1.1% vs. 4.0%, *P* < .001). The discrimination performance of the present model for high-risk patients with advanced CRN was better than that of the Asia-Pacific Colorectal Screening score (AUC = 0.678, *P* < .001) and Schroy’s CAN index (AUC = 0.672, *P* < .001).

**Conclusion:**

The present 5-item risk model can be calculated readily using a simple questionnaire and can identify the low- and high-risk groups of advanced CRN at the first screening colonoscopy. This model may increase colorectal cancer risk awareness and assist healthcare providers in encouraging the high-risk group to undergo a colonoscopy.

## Introduction

Colorectal cancer (CRC) is the third most common cancer in the world [1]. A colonoscopy is considered the preferred CRC screening modality [2]; however, adherence is generally not sufficient [3]. One of the barriers to CRC screening is a lack of perceived risk among the patients and primary care providers [4]. Risk stratification provides a rational strategy for facilitating appropriate CRC screening and can improve the distribution of resources. A prerequisite for this risk stratification approach is the accessibility of a precise risk assessment tool.

Although several risk prediction models for screening target lesions, CRC, or advanced colorectal neoplasia (CRN) have been developed [4–14], previous models have some limitations, such as the lack of inclusion of possible risk factors [4, 5, 7, 9–11, 13]. In the current healthcare system, electronic medical records (EMRs) encompass a plethora of data related with patients, such as demographics, vital signs, medical history, medication, laboratory test results, results from laboratory and imaging studies. The use of medical data mining and correlational studies using EMRs could serve as a valuable resource to aid the determination of unrevealed risk factors under deductive assumptions to establish a real-world prediction model for advanced CRN.

However, EMR data contains unstructured data, such as endoscopic and pathology reports, which requires laborious efforts for transforming text to numerical data [15]. Recent advanced natural language processing algorithms, such as the Concept Extraction-based Text Analysis System (CETAS), are able to transform information from endoscopic and pathology reports to a numerical dataset. In this study, we constructed a database using the EMR data from 49,450 patients who underwent their first screening colonoscopy as part of routine health check-up examinations by means of the CETAS and developed a risk prediction model to identify individuals at high risk of advanced CRN.

## Methods

### Study population

The Center for Health Promotion of Samsung Medical Center, Seoul, Republic of Korea, provides regular health screening examinations that include a colonoscopy [16]. We included consecutive subjects who underwent a screening colonoscopy during health screening examinations at the Center for Health Promotion between January 2003 and December 2012. Regular routine health screening is very common in Korea due to the Industrial Safety and Health Law [16, 17]. The health screening examinations were performed as described previously [16]. All participants completed a questionnaire and received a detailed physical examination as part of the screening program. Self-administered questionnaire data were used to identify current smoking status, alcohol drinking frequency, physical activity, family history of colon cancer, history of colorectal polyps/cancer, comorbidities, and regular use of aspirin. Participants were asked to fast for at least 12 hours and to avoid smoking on the morning of the examination. Blood samples were collected on the day of the colonoscopy. Serum biochemical tests were carried out using an automatic analyzer at the Department of Laboratory Medicine at Samsung Medical Center.

### Screening colonoscopies

All colonoscopies were performed by board-certified endoscopists. During colonoscopy, the location, size, number, and appearance of CRN were recorded. The location was assessed by the endoscopists, and the size was estimated using open biopsy forceps. The gross appearance of each lesion was classified using Paris endoscopic classification [18]. All of the colorectal lesions were histologically evaluated and classified according to the World Health Organization classification [19]. However, because the colonoscopy and pathology reports were described by the performing endoscopists and pathologists, the natural language for describing the lesions was different in each report despite using standardized terms. For example, even though the information was the same, the endoscopists used different units, such as cm or mm, and various modifiers, such as elevated, raised, upraised, protruded, and bulged. Therefore, the reports were considered to contain unstructured data, and it was difficult to extract unified forms of variables in real practice.

### Data collection

This study used only de-identified medical records that were collected for administrative or clinical purposes as part of routine health screening examinations in the Center for Health Promotion of Samsung Medical Center. The Center for Health Promotion provides researchers de-identified information for biomedical research, which was approved by the Institutional Review Board of Samsung Medical Center for studies that investigate decision-making and the relationships and potential patterns between disease progression and management. The EMRs included both structured and unstructured data. Structured data refer to information that was organized in a row-column database including demographics, physical measurement, smoking, alcohol drinking, physical activity, co-morbidities, aspirin use, and laboratory biochemical measurements. Unstructured data refers to information that does not reside in a traditional row-column database including the free text from colonoscopy and pathology reports. This study was approved by the Institutional Review Board of Samsung Medical Center, which waived the requirement for informed consent because the researchers only obtained de-identified routinely collected data from the institution's clinical data warehouse.

### Unstructured text data analysis: Concept Extraction-based Text Analysis System (CETAS)

**Fig 1:**
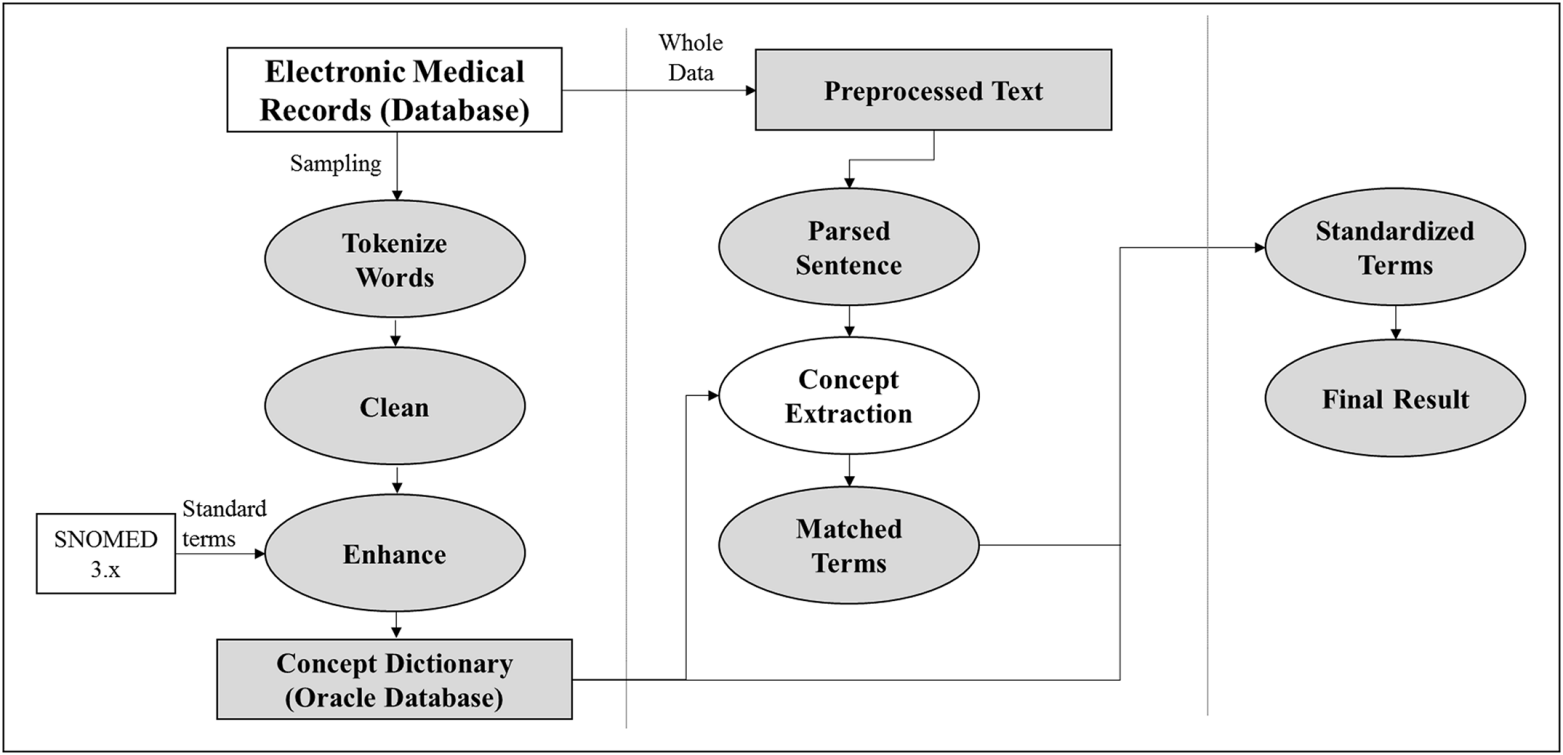
Process diagram of a Concept Extraction-based Text Analysis System.

Among the data collected in this study, we obtained the data about the number and size related to CRN from the free text of the colonoscopy reports, and the histology and dysplasia grade related to CRN from the free text of the pathology reports. Unstructured data were transformed from text to numerical data by the CETAS. The CETAS is based on SAS Enterprise Contents Categorization 12.2 (SAS Institution; Cary, NC, USA), and it does not have add-on modules, such as text mining. SAS ECC 12.2 is an NLP solution that is separate from SAS Base; since it has a built-in LITI (Language Interface Taxonomy Interface) for performing Concept Extraction in a simple and effective manner in the text, it offers a solution that enables rule-based construction of the matching of terms and extraction (Fig 1) [15, 20].

#### 1. Concept dictionary

In order to create a Concept Dictionary that underlies the configuration of the Concept Extraction Rule, by extracting about 500 colonoscopy tests and pathologic result reports in a random sampling manner, the terms that represent the information for the colorectal polyps which is the number, location, size, histology, dysplasia grade through a natural language processing methodology are organized and cleansed. In this process, in order to determine, the standard terminology of non-standard terms included in the EMR database, a Concept Dictionary was configured by referencing the SNOMED 3.x.

#### 2. Preprocess

**Fig 2:**
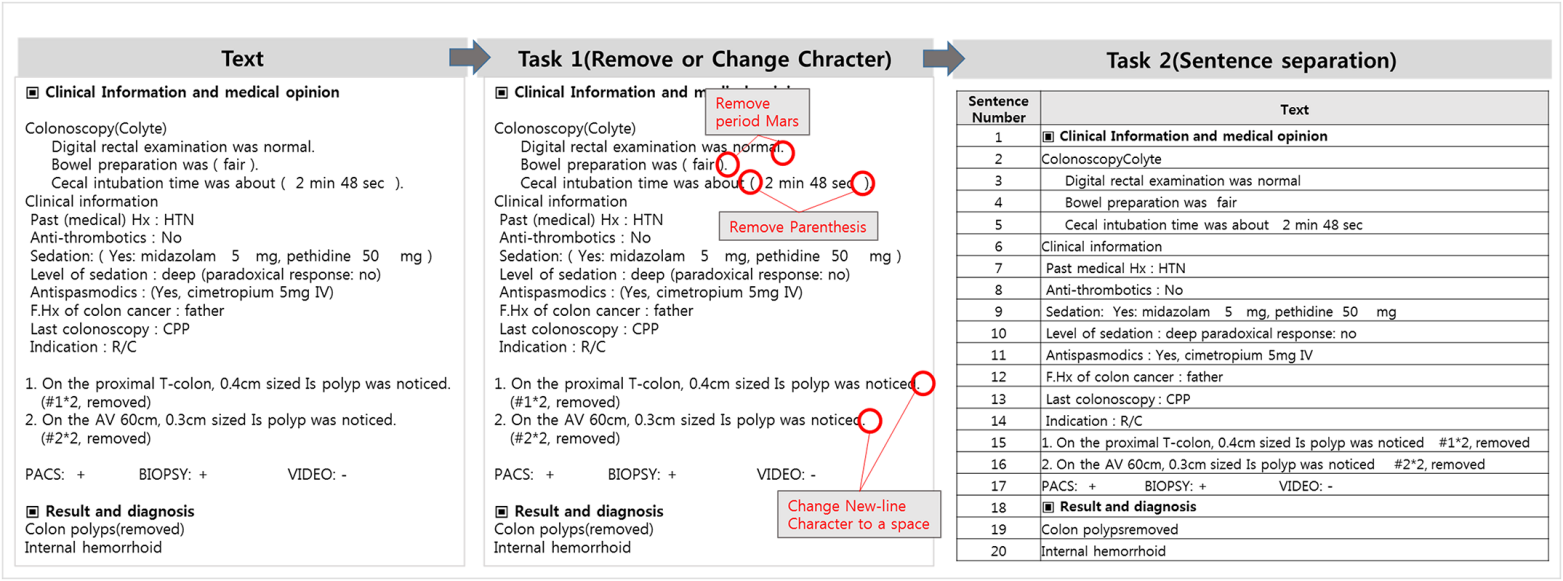
Text pre-processing.

A pretreatment process for changing the colonoscopy reports created in different sentence structures by each endoscopists into coherent sentence structures is constructed (Fig 2). The Preprocessing is comprised of two operations. Task1 is composed of functions that delete or change the special symbols that became non-standardized special symbols, such as Bullet Mark, comma, line breaks, spacing, etc.; through which, a base that can perform Concept Extraction based on the special symbols in the natural language processing steps was created. Task2 performs the task of dividing the text into sentences or syntactical units in accordance with the period, line breaks, conjunctions, prepositions, etc. With this, in the case where the text about organs and lesions is expressed through a number of sentences and paragraphs, the error of generating results in conjunction with the information of sentence 1 and sentence 2 that are not related in terms of processing the natural language can be reduced.

#### 3. Concept extraction

**Fig 3:**
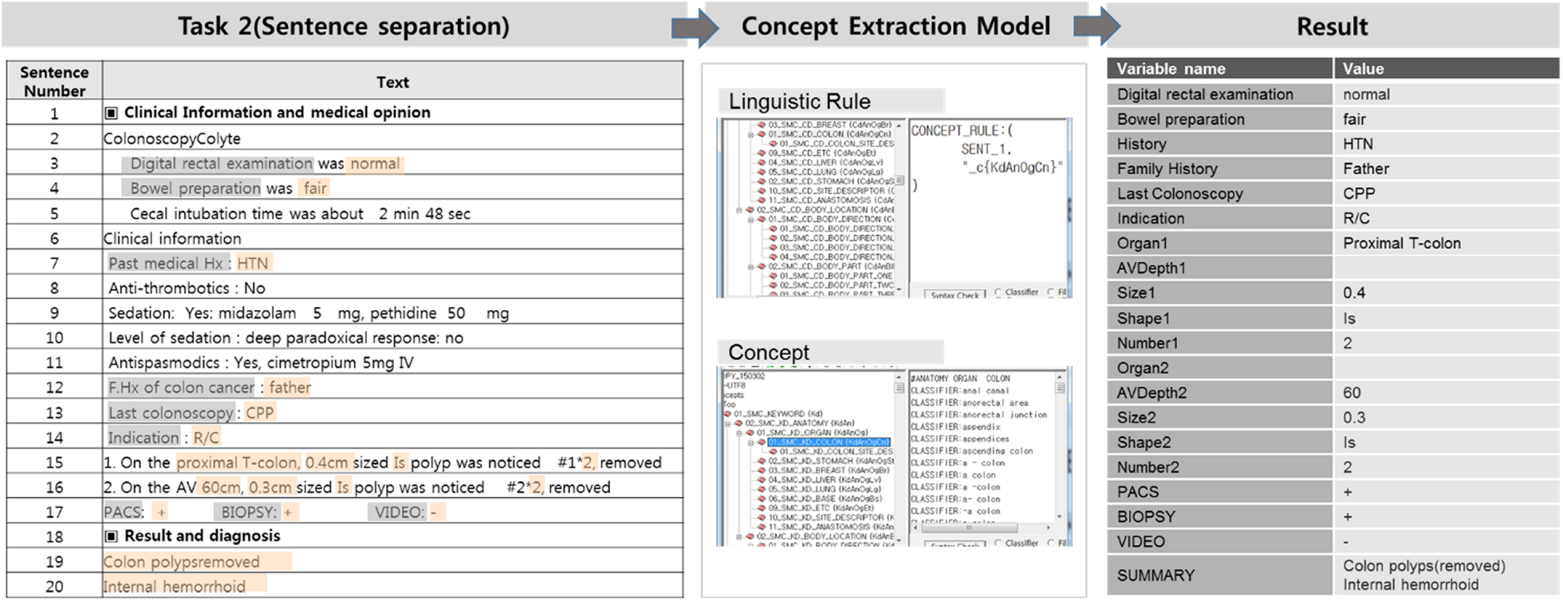
Concept extraction process.

As a natural language processing step, categories are configured in accordance with the hierarchical structure of the colorectal structures and lesions, and the Concept Extraction Rule was developed for each category. The effectiveness for Rule-based natural language processing has already been frequently proven in the previous studies.[21, 22] Concept Extraction is performed through the Rule that extracts the terms stored in the Concept Dictionary that is within the particular words expressed in sentences or within the Keyword Count specified by special symbols. As such, the researchers did not have to change the Rule when adding/removing terms; they simply had to change the Concept Dictionary and that automatically changed the results of the Concept Extraction (Fig 3).

#### 4. Validation

Through a random sampling of 500 colonoscopy and pathology result reports from the clinical data warehouse of Samsung Medical Center, the first ever Concept Extraction Rule was created, and by expanding the sample size to 2,000, the precision of the Rule was increased. After the Concept Extraction Rule was created, the accuracy was verified by comparing the Concept Extraction results and the results manually generated. The accuracy can be verified by the following two indicators.

Precision= Correct(Correct+Missed)

Recall= Correct(Correct+Incorrect)

Precision means the ability to find the correct data when it is in the colonoscopy and pathology result reports, and Recall means the ability to find only the correct data when it is in the colonoscopy and pathology result reports to the meaning. In order to verify the accuracy of the Concept Extraction Rule built in the CETAS, finally, Precision and Recall were calculated through a random sampling of 50 specimens from the 3 months, and the Precision and Recall were each 99.27% and 99.83%, respectively (Table 1). After validation, unstructured data from remaining 48950 colonoscopy and pathology was transformed text to numerical data.

**Table 1:** Comparison of concept extraction results and manual data extraction.

Category	Precision (%)	Recall (%)	Category	Precision (%)	Recall (%)
PAST MEDICAL HISTORY	100.00	100.00	LESION	100.00	100.00
ANTITHROMBOTICS	100.00	100.00	ABNORMALITY	100.00	100.00
FAMILY HISTORY OF CANCER	100.00	100.00	HISTOLOGICAL CLASSIFICATIONADJ	100.00	100.00
LAST COLONOSCOPY	100.00	100.00	HISTOLOGIC TYPE	100.00	100.00
INDICATION	100.00	97.75	TUMOR GRADING	100.00	100.00
SEDATION	100.00	100.00	SIZE	92.05	98.78
MIDAZOLAM	100.00	100.00	NUMBER	100.00	100.00
PETHIDINE	100.00	100.00	SHAPE	100.00	100.00
LEVEL OF SEDATION	100.00	98.88	COLOR	100.00	100.00
PARADOXICAL RESPONSE	100.00	100.00	VIDEO	95.51	100.00
ANTISPASMODICS	100.00	100.00	SLIDE	95.51	100.00
CIMETROPIUM	100.00	100.00	ORGAN	100.00	100.00
DIGITAL RECTAL EXAMINATION	96.63	100.00	BIOPSY	100.00	100.00
BOWEL PREPARATION	100.00	100.00	BIOPSY STATUS	98.88	100.00
CECAL INBUTIONTIME	100.00	97.75	BIOPSY METHOD	93.26	100.00
WITHDRAWAL TIME	100.00	97.75	SUBMUCOSAL INJECTION	100.00	100.00
INSERTED UPTO	100.00	98.88	HEMOSTASIS	100.00	100.00
ORGAN	100.00	100.00	DIAGNOSIS	100.00	100.00
SITE	98.88	100.00	IMPRESSION	100.00	100.00
			**Total**	**99.27**	**99.83**

### Study design

We performed a cross-sectional analysis of patients ≥ 20 years of age who underwent their first screening colonoscopy. The exclusion criteria were as follows: 1) incomplete colonoscopy, 2) poor (semisolid stool that could not be suctioned or washed away and less than 90% of surface seen) and inadequate (repeat preparation and colonoscopy needed) bowel preparation, 3) incomplete colonoscopy report about the number and size related to CRN, 4) incomplete pathology report about the histology and dysplasia grade related to CRN, 5) history of previous colonoscopy, 6) history of colorectal polyps, cancer, or surgery, and 7) inflammatory bowel disease.

### Definition of outcome measurement

An advanced CRN was defined as a cancer or adenoma that was at least 10 mm in diameter and had high-grade dysplasia, villous or tubulovillous histological characteristics, or any combination thereof [23]. For patients with multiple neoplasms, the size and appearance of the neoplasms with advanced pathology or of the largest polyp were reported. The main outcome measurement in this study is an advanced CRN detected by means of a colonoscopy and evaluated pathologically.

### Prediction model

Structured data and unstructured data transformed from text to numerical data using the CETAS were used as the input variables of the prediction model. The enrolled subjects were randomly partitioned into a training set and a validation set using a 50–50 allocation. Candidate predictors with *P* < .10 in univariate analyses were included in the multivariable logistic regression. Backward selection was used to remove variables with not significant (*P* < .05) contributions to the multivariable model fit. Two prediction models were fitted. The first one used both inquiry and lab variables, and the second only used inquiry variables.

### Model performance and calibration

A two-sided alpha of 5% was used as insertion and deletion criteria of the two-stage variable selection in fitting a prediction model (i.e., training). The prediction score from the fitted prediction model was applied to the validation set, and the performance of the prediction model was evaluated using area under receiver operating curve (AUC) analysis. Models with a AUC near 1 suggest excellent predictive ability, and an AUC near 0.5 indicates hardly any predictive ability. The calibration is a measure of how accurately the predicted probabilities of advanced CRN inferred from the training set match the subsequently observed event rate in the validation set. The negative predictive value (NPV) is the probability that a patient who is termed “no disease” by the risk score really has no disease. We want this probability to be very high (at least 99%) so as not to miss any significant disease. A cutoff value for the trained risk score was identified and shown to have over 99% negative predictive value when applied to the test set and the combined data set.

## Results

### Study population

**Fig 4:**
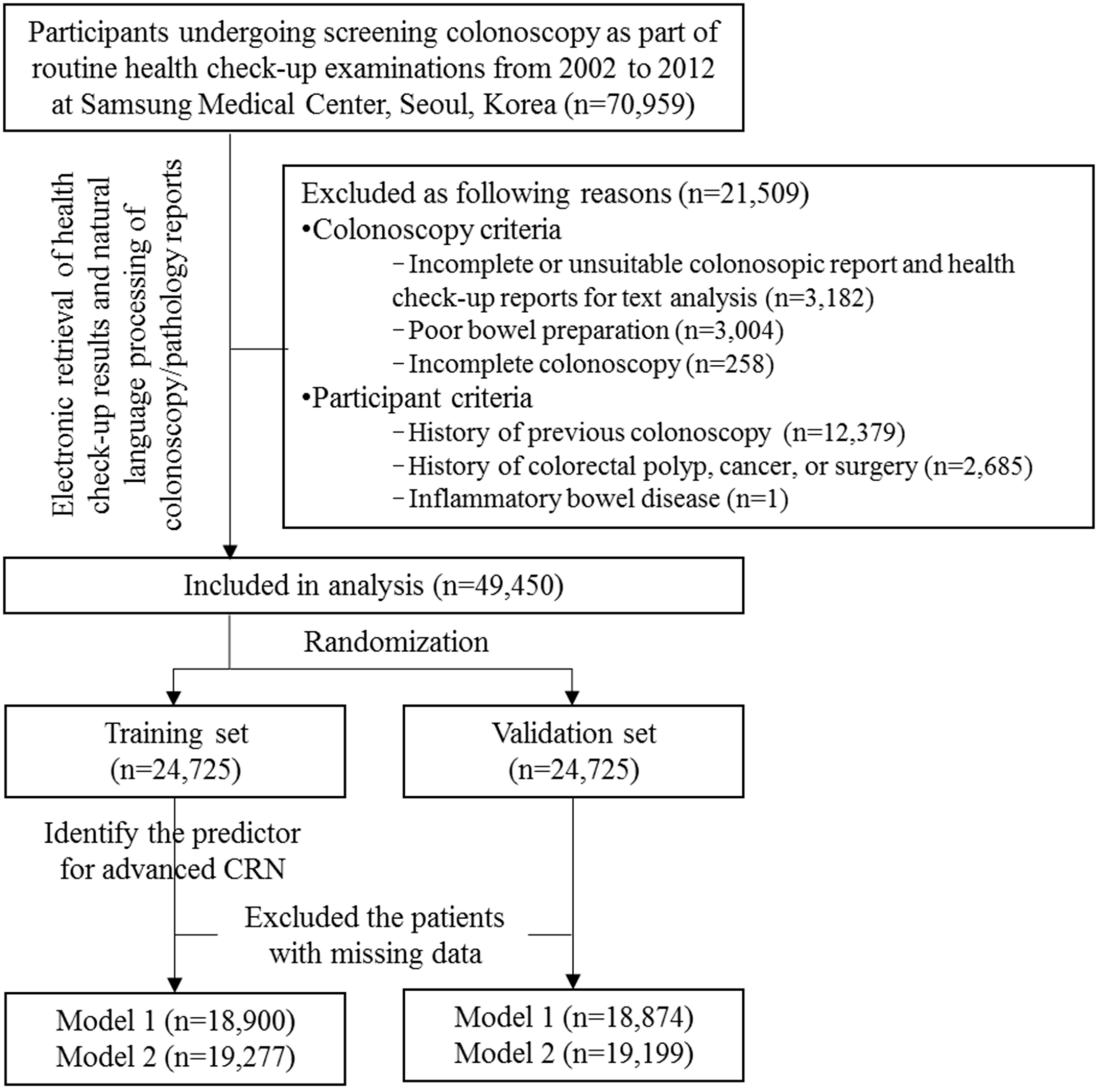
Flow diagram of the study population.

A total of 70,959 consecutive subjects underwent screening colonoscopy during health screening examinations at the Center for Health Promotion. We excluded 21,509 subjects who had incomplete or unsuitable reports for text analysis; poor bowel preparation; incomplete colonoscopy; or history of previous colonoscopy, colorectal polyps, cancer, or surgery, or inflammatory bowel disease. For subjects who underwent multiple colonoscopies, we selected the first colonoscopy for the present analysis. Finally, this study used only de-identified data from 49,450 participants who underwent their first screening colonoscopy and a health check-up. A flow diagram of the study population is shown in Fig 4. Of the eligible 49,450 patients who underwent their first screening colonoscopy, 27,688 were male (55.99%) and 21,762 were female (44.01%), all were Korean, and the mean age was 49.86 ± 9.33 years. One or more colorectal adenomas were found in 14,716 (29.8%) patients, 1,025 (2.1%) of whom had advanced adenoma, and 92 of whom had invasive cancer (0.2%). The overall prevalence of advanced CRN was 2.3%. The clinical characteristics of the enrolled participants are listed in Table 2. Enrolled participants were randomly divided into training and validation sets using a 50–50 allocation.

**Table 2:** Clinical characteristics of enrolled subjects.

Variable	Total		Training set		Validation set		*p*
	N	Value	N	Value	N	Value	
Demographics							
Age (years), mean ± SD	49,450	49.9 ± 9.3	24,726	49.9 ± 9.4	24,724	49.8 ± 9.3	0.160
Sex	49,450		24,726		24,724		0.008
Female, n (%)		21,762 (44.0)		10,735 (43.4)		11,027 (44.6)	
Male, n (%)		27,688 (56.0)		13,991 (56.6)		13,697 (55.4)	
Family history of colorectal cancer	45,583		22,759		21,706		0.694
Yes, n (%)		2,251 (4.9)		1,133 (5.0)		1,118 (4.9)	
No, n (%)		43,332(95.1)		21,626 (95.0)		21,706 (95.1)	
Physical measurement							
Body mass index, mean ± SD	44,581	23.7 ± 3.1	22,275	23.7 ± 3.1	22,306	23.6 ± 3.1	0.410
Waist circumference (cm), mean ± SD	44,145	83.4 ± 44.3	22,057	83.7 ± 62.0	22,088	83.2 ± 9.1	0.229
Body fat percentage* (%), mean ± SD	49,058	25.5 ± 6.6	24,517	25.4 ± 6.5	24,541	25.5 ± 6.6	0.813
Cigarette smoking							
Smoking status	42,579		21,271		21,308		0.319
Non-smoker, n (%)		23,841 (56.0)		11,838 (55.7)		12,003 (56.4)	
Ex-smoker, n (%)		5,260 (12.3)		2,631 (12.3)		2,629 (12.3)	
Current smoker, n (%)		13,478 (31.7)		6,802 (32.0)		6,676 (31.3)	
Smoking duration (year), mean ± SD	43,108	9.9 ± 12.8	21,526	10.0 ± 12.9	21,582	9.8 ± 12.7	0.143
Smoking amount (pack/day), mean ± SD	43,107	0.9 ± 1.1	21,534	0.9 ± 1.1	21,573	0.9 ± 1.1	0.238
Alcohol drinking							
Regular alcohol drinking	43,777		21,868		21,909		0.719
Yes, n (%)		19,814 (45.3)		9,879 (45.2)		9,935 (45.4)	
No, n (%)		23,963 (54.7)		1,1989 (54.8)		11,974 (54.6)	
Drinking duration (year), mean ± SD	26,395	23.8 ± 10.1	13,250	23.8 ± 10.2	13,145	23.8 ± 10.0	0.551
Drinking frequency (/week), mean ± SD	40,171		20,096		20,075		
No drinking, n (%)		19,814 (49.3)		9,879 (49.2)		9,935 (49.5)	0.843
Once a week, n (%)		3,268 (8.1)		1,671 (8.3)		1,597 (8.0)	
2–3 times per month, n (%)		5,792 (14.4)		2,897 (14.4)		2,895 (14.4)	
1–2 times per week, n (%)		6,730 (16.8)		3,344 (16.6)		3,386 (16.9)	
3–4 times per week, n (%)		3,436 (8.6)		1,737 (8.6)		1,699 (8.5)	
5–6 times per week, n (%)		779 (1.9)		411 (2.0)		368 (1.8)	
Everyday, n (%)		352 (0.9)		157 (0.8)		195 (1.0)	
Drinking amount at one (bottle), mean ± SD	40,027	1.2 ± 1.4	20,028	1.2 ± 1.4	19,999	1.2 ± 1.4	0.782
Physical activity							
Type of physical activities†	29,150		14,558		14,592		0.132
Strenuous activities, n (%)		2,148 (7.4)		1,097 (7.5)		1,051 (7.2)	
Moderate activities, n (%)		6,719 (23.1)		3,299 (22.7)		3,420 (23.4)	
Mild activities, n (%)		17,895 (61.4)		8,930 (61.3)		8,965 (61.4)	
None, n (%)		2,388 (8.2)		1,232 (8.5)		1,156 (7.9)	
Physical activity frequency (/week), mean ± SD	28,510	2.8 ± 0.9	14,267	2.79 ± 0.88	14,243	2.80 ± 0.87	0.121
Physical activity duration (minutes), mean ± SD	28,663	36.8 ± 11.6	14,337	36.74 ± 11.62	14,326	36.82 ± 11.49	0.562
Co-morbidity							
Hypertension, n (%)		6,545 (13.2)		3,325 (13.5)		3,220 (13.0)	0.166
Diabetes, n (%)		1,917 (3.9)		932 (3.8)		985 (5.0)	0.216
Hyperlipidemia, n (%)		1,941 (3.9)		932 (3.8)		1,009 (4.1)	0.355
Aspirin use							
Regular use, n (%)		2,612 (5.3)		1,336 (5.4)		1,276 (5.2)	0.229
No use, n (%)		46,838 (94.7)		23,390 (94.6)		23,448 (94.8)	
Laboratory measurement							
Hemoglobin, mean ± SD	49,136	14.3 ± 31.5	24,556	14.3 ± 1.6	24,580	14.3 ± 1.5	0.601
Hematocrit, mean ± SD	49,136	42.4 ± 34.2	24,556	42.4 ± 4.2	24,580	42.4 ± 4.2	0.463
Platelet, mean ± SD	49,136	234.9 ± 52.3	24,556	234.7 ± 51.9	24,580	235.1 ± 52.8	0.421
Prothrombine time (INR)	46,820	1.0 ± 0.1	23,404	1.0 ± 0.1	23,416	1.0 ± 0.1	0.537
Total_protein	49,137	7.1 ± 0.4	24,559	7.1 ± 0.4	24,578	7.1 ± 0.4	0.051
Albumin	49,137	4.5 ± 0.3	24,559	4.5 ± 0.3	24,578	4.5 ± 0.3	0.419
Total bilirubin, mean ± SD	49,137	0.9 ± 0.4	24,559	0.9 ± 0.4	24,578	0.9 ± 0.4	0.507
Aspartate transaminase	49,142	26.1 ± 16.1	24,561	26.2 ± 16.6	24,581	26.0 ± 15.6	0.233
Alanine transaminase	49,142	26.4 ± 24.6	24,561	26.5 ± 24.6	24,581	26.3 ± 24.5	0.292
Alkaline phosphatase	49,137	63.3 ± 18.3	24,558	63.6 ± 18.7	24,579	62.9 ± 17.9	0.001
γ-glutamyltransferase, mean ± SD	48,603	33.9 ± 44.8	24,295	34.1 ± 47.0	24,308	33.7 ± 42.6	0.268
Uric acid, mean ± SD	49,129	5.2 ± 1.4	24,554	5.2 ± 1.4	24,575	5.2 ± 1.4	0.001
Blood urea nitrogen	49,135	13.3 ± 3.4	24,559	13.3 ± 3.4	24,576	13.3 ± 3.4	0.499
Creatinine	49,138	0.9 ± 0.2	24,560	0.9 ± 0.2	24,578	0.9 ± 0.2	0.182
Fasting glucose, mean ± SD	49,146	93.7 ± 18.0	24,563	93.7 ± 17.7	24,583	93.8 ± 18.3	0.625
Hemoglobin a1c, mean ± SD	47,575	5.6 ± 0.7	23,790	5.6 ± 0.7	23,785	5.6 ± 0.7	0.916
Insulin	34,601	7.4 ± 4.4	17,337	7.4 ± 4.5	17,264	7.3 ± 4.3	0.206
C-peptide	34,602	1.7 ± 0.8	17,337	1.7 ± 0.8	17,265	1.7 ± 0.8	0.174
Total cholesterol, mean ± SD	49,153	196.5 ± 34.7	24,569	196.7 ± 34.8	24,584	196.3 ± 34.5	0.194
Triglyceride, mean ± SD	48,757	119.0 ± 76.0	24,377	119.2 ± 75.6	24,380	118.7 ± 76.5	0.501
HDL-cholesterol, mean ± SD	48,755	55.9 ± 14.6	24,376	55.4 ± 14.7	24,379	55.6 ± 14.6	0.120
LDL- cholesterol, mean ± SD	48,759	123.9 ± 31.1	24,378	124.2 ± 31.2	24,381	123.7 ± 31.0	0.117
C-reactive protein, mean ± SD	43,613	0.1 ± 0.3	21,800	0.1 ± 0.3	21,813	0.1 ± 0.3	0.073
Calcium, mean ± SD	49,128	9.2 ± 0.4	24,554	9.2 ± 0.4	24,574	9.2 ± 0.4	0.755
Ferritin, mean ± SD	40,343	121.1 ± 120.2	20,211	122.4 ± 126.5	20,132	119.7 ± 113.6	0.089
Colonoscopic and pathologic finding of enrolled patients							
No adenoma	34,734	70.2%	17,377	70.3%	17,357	70.2%	0.855
Serrated polyp	5,868	11.9%	2,916	11.8%	2,952	11.9%	0.614
Any adenoma	14,716	29.8%	7,349	29.7%	7,367	29.8%	0.855
Number of adenomas							
1 or 2	12,251	24.8%	6,084	24.6%	6,167	24.9%	0.319
≥3	2,465	5.0%	1,265	5.1%	1,200	4.9%	
Size of the largest adenoma							
≤10 mm	14,002	28.3%	6,994	28.3%	7,008	28.3%	0.976
>10 mm	714	1.4%	355	1.4%	359	1.5%	
Histology of adenoma							
Tubular adenoma	14,586	29.5%	7,279	29.4%	7,307	29.6%	0.659
Tubulovillous or villous adenoma	130	0.3%	70	0.3%	60	0.2%	
Dysplasia grade							
Low-grade dysplasia	14,511	29.3%	7,251	29.3%	7,260	29.4%	0.928
High-grade dysplasia	125	0.3%	59	0.2%	66	0.1%	
Non-advanced adenoma	13,691	27.7%	6,836	27.7%	6,855	27.7%	0.981
Advanced adenoma^§^ ^-^	989	2.0%	495	2.0%	494	2.0%	0.975
Invasive cancer	92	0.2%	45	0.2%	47	0.2%	0.834
Advanced neoplasia^¶^	1,025	2.1%	513	2.1%	512	2.1%	0.976

*Measured by bioelectrical impedance device

^†^Type of physical activities have done for the last 7 days including recreation, exercise, sports activities, activities at the work

- strenuous activities—ex) labor, aerobics, fast running bicycle, jogging, soccer- moderate activities—ex) a quick step, swimming, mountain climbing, four-up tennis- mild activities—ex) walking, golf, household-chores- none—I do not even walk for 10 m

^§^Advanced adenoma was defined as adenoma with villous histology, high-grade dysplasia, or size >10 mm

^¶^Advanced neoplasia was referred to advanced adenoma and invasive cancer

### Identifying risk predictors and developing a candidate risk prediction model

To identify the patients with advanced CRN among the individuals who underwent their first colonoscopy, a stepwise logistic regression using all available variables listed in Table 1 was conducted for the imputed training set. We identified age, gender, diabetes, aspirin use, smoking duration, alcohol drinking frequency, drinking duration, uric acid, and γ-glutamyltransferase as the potential predictors (Table 3). Predictors for advanced CRN were refined using the complete data from the training set and excluded drinking duration and uric acid due to *P*-values > 0.3. Finally, age, gender, smoking duration, alcohol drinking frequency, aspirin use, and γ-glutamyltransferase were included in the prediction model (model 1). The prediction score from the refined prediction model 1 was determined by the following equation:

$$\begin{array}{l} Advanced\;CRN\;prediction\;score\left(model\;1\right)\;=-8.3317+0.00149\times \gamma -Glutamyltransferase\;\\ +0.0149\times Smoking\;duration\;\left(year\right)\;+0.0943\times Drinking\;frequency\text{ (}no\;drinking\;=\;0\;/\;\\ once\;a\;week\;=\;1\;/\;2-3\;times\;per\;month\;=\;2\;/\;1-2\;times\;per\;week\;=\;3\;/\;3-4\;times\;per\;\\ week\;=\;4\;/\;5-6\;times\;per\;week\;=\;5\;/\;everyday\;=\;6)\;-\;0.572\;\times Aspirin\;use\text{ (}regular\;use\;=\;\\ 1\;/\;no\;use\;=\;0)\;+0.3801\;\times \;Gender\left(male\;=\;1\;/\;female\;=\;0\right)\;+0.0738\times Age\;\left(years\right) \end{array}$$

**Table 3:** Stepwise logistic regression for predicting patients with advanced colorectal neoplasia among individuals who underwent their first colonoscopy.

1. Predictor selection for advanced neoplasia using the imputed training set			
Parameter	Estimate	Standard Error	*P*
Intercept	-8.282	0.394	< .001
Uric acid	0.062	0.039	.110
γ-Glutamyltransferase	0.001	0.001	.035
Smoking duration	0.015	0.004	< .001
Drinking duration	0.010	0.007	.131
Drinking frequency	0.082	0.030	.007
Aspirin use	-0.299	0.096	.002
Diabetes	0.225	0.110	.041
Gender	0.122	0.069	.075
Age	0.065	0.006	< .001
2. Predictor refining for advanced neoplasia using the complete training set: variables with a *P*-value > 0.3 were excluded			
Parameter	Estimate	Standard Error	*P*
Intercept	-8.720	0.515	< .001
Uric acid	0.073	0.049	.140
γ-Glutamyltransferase	0.001	0.001	.026
Smoking duration	0.015	0.005	.002
Drinking duration	0.005	0.009	.538
Drinking frequency	0.089	0.035	.011
Aspirin use	-0.192	0.111	.082
Gender	0.138	0.123	.261
Age	0.071	0.009	< .001
Parameter	Estimate	Standard Error	*P*
Intercept	-8.710	0.432	<001
Uric acid	0.050	0.044	.253
γ-Glutamyltransferase	0.001	0.001	.024
Smoking duration	0.015	0.004	.001
Drinking frequency	0.095	0.031	.002
Aspirin use	-0.288	0.104	.006
Gender	0.153	0.083	.064
Age	0.074	0.006	< .001
3. Prediction model for advanced neoplasia (Model 1)			
Parameter	Estimate	Standard Error	*P*
Intercept	-8.428	0.354	< .001
γ-Glutamyltransferase	0.002	0.001	.016
Smoking duration	0.015	0.004	.001
Drinking frequency	0.094	0.031	.002
Aspirin use	-0.286	0.104	.006
Gender	0.190	0.076	.012
Age	0.074	0.006	< .001
Parameter	Estimate	Standard Error	*P*
Intercept	-8.390	0.350	<. 001
Smoking duration	0.015	0.004	< .001
Drinking frequency	0.100	0.031	.001
Aspirin use	-0.289	0.104	.006
Gender	0.205	0.075	.007
Age	0.074	0.006	< .001

Among the identified predictors, γ-glutamyltransferase was the only laboratory parameter that requires blood sampling and laboratory costs. When γ-glutamyltransferase was removed from the prediction model, all predictors could be obtained from a simple questionnaire, and a simple 5-item risk index could be readily determined from the questionnaire clinical data. The final prediction model was constructed with age, gender, smoking duration, alcohol drinking frequency, and aspirin use (model 2). The prediction score from the refined prediction models 1 and 2 was determined by the following equation:

$$\begin{array}{l} AdvancedCRNpredictionscore\left(Model2\right)=-8.39+0.0154\times Smokingduration\left(year\right)\\ +0.1003\times Drinkingfrequency(nodrinking=0/onceaweek=1/2-3timesper\\ month=2/1-2timesperweek=3/3-4timesperweek=4/5-6timesperweek=\\ 5/everyday=6))-0.5772\times Aspirinuse\left(regularuse=1/nouse=0\right)+0.4098\times \\ Gender\left(male=1/female=0\right)+0.0736\times Age\left(years\right) \end{array}$$

### Evaluating the performance of the prediction model

**Fig 5:**
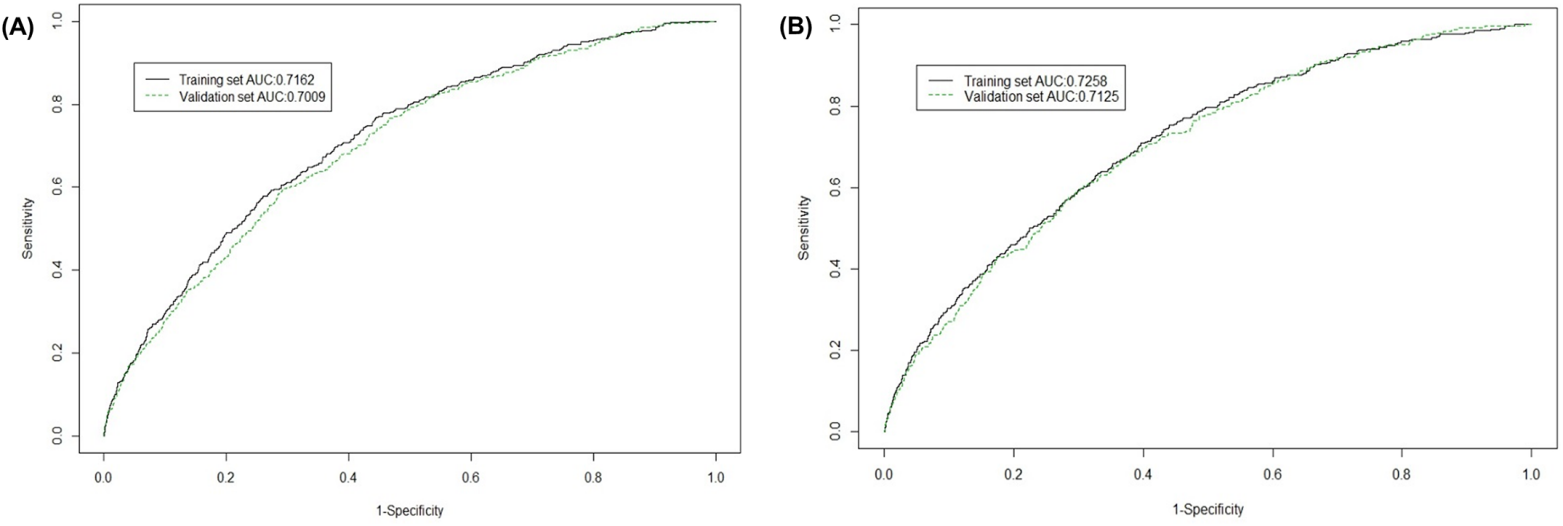
Model performance. Area under the receiver operating curve (AUC) was calculated to evaluate the discrimination power between the training set (line) and validation set (dot) in prediction model 1 (A) and model 2 (B).

Discrimination refers to the ability to separate the variables with events from those without events. Using the prediction models 1 and 2, AUC values were calculated and used to evaluate the discrimination power of the prediction models. The AUC for prediction model 1 was 0.716 for the training set and 0.701 for the validation set (Fig 5A), whereas the AUC for prediction model 2 was 0.726 for the training set and 0.713 for the validation set (Fig 5B). Model 2 showed slightly higher discriminatory ability than model 1, although the risk factors were eliminated. The reason why model 2 was superior to model 1 was that the number of participants included in the calculation was larger in model 2 (training set: n = 18,874, validation set: n = 19,199) than model 1 (training set: n = 18,900, validation set: n = 19,277).

**Fig 6:**
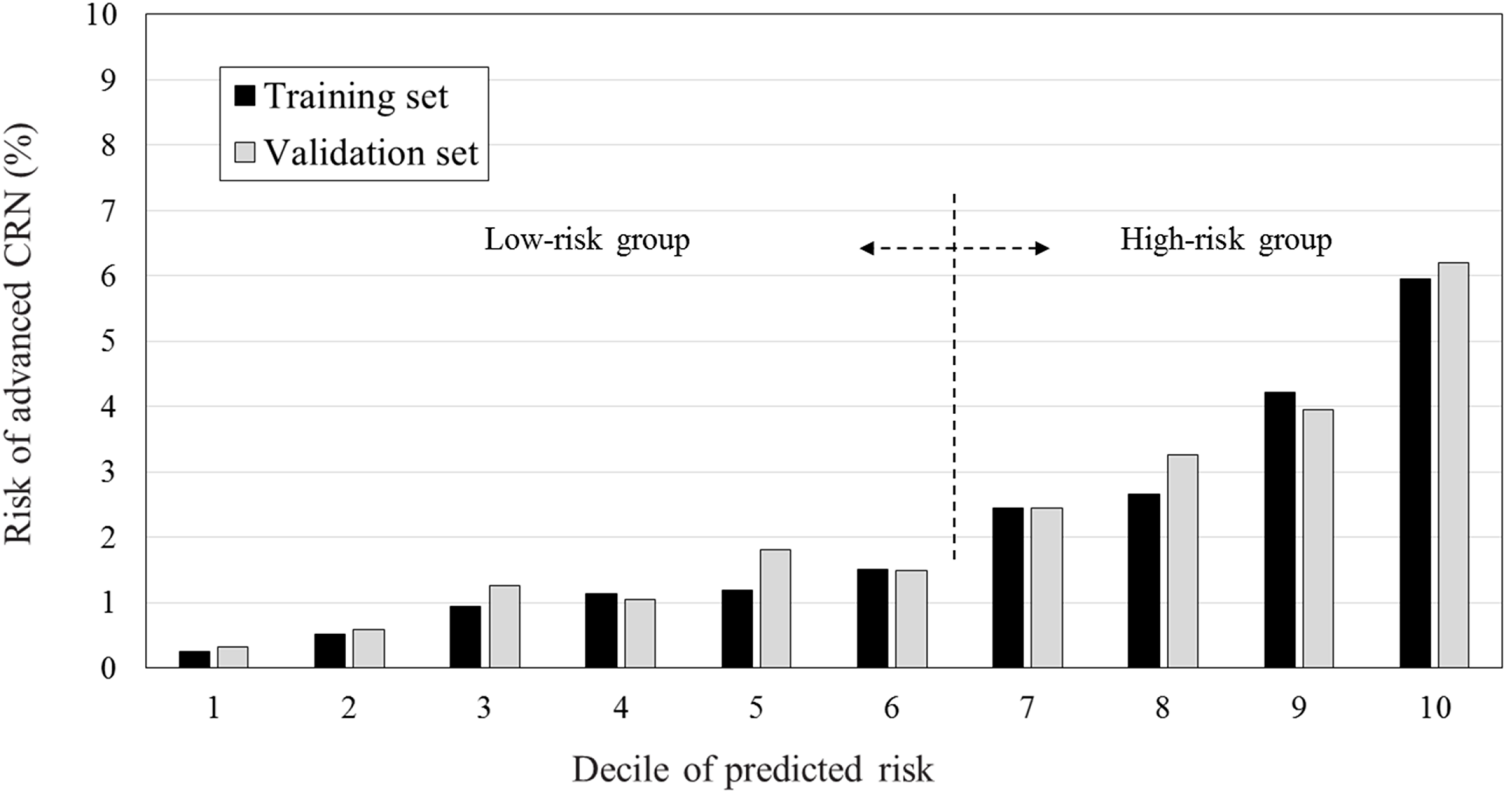
Model calibration. Cut-off values to discriminate between the high- and low-risk groups for advanced colorectal neoplasia were set at the point between the sixth and seventh deciles based on the risk of advanced colorectal neoplasia.

Prediction model 2 was selected as the final prediction model for advanced CRN. The calibration is a measure of how accurately the predicted probabilities of advanced CRN inferred from the training set match the subsequently observed event rate in the validation set. The individuals included in the training set were divided into deciles according to predicted risk for advanced CRN. Then, the predicted rate of the training set and observed rates of the validation set in each category were compared (Fig 6), indicating good calibration performance. To improve clinical utilization, cut-off values were set at the point of discrimination between the high- and low-risk group for advanced CRN in simulated calibration charts. Between the sixth and seventh deciles, the risk of advanced CRN increased from 1.51% to 2.45% in the training set and 1.50% to 2.45% in the validation set. The cut-off value of -4.195 was set at this point between the sixth and seventh deciles (Table 4).

**Table 4:** Model calibration and estimation of cut-off value for discrimination between high- and low-risk for advanced colorectal neoplasia (CRN).

Decile of predicted risk	Training set		Validation set			Risk group
	N	Prevalence of advanced CRN (%)	N	Prevalence of advanced CRN (%)		
1	1922	0.260	1901	0.316	1.067	Low-risk group
2	1922	0.520	2064	0.581		
3	1922	0.937	1983	1.261		
4	1922	1.145	2079	1.058		
5	1922	1.197	1715	1.808		
6	1922	1.509	1871	1.497		
**7**	1922	2.445	1960	2.449	3.955	High-risk group
8	1922	2.653	1867	3.267		
9	1922	4.214	1873	3.951		
10	1929	5.962	1885	6.207		

### Discrimination of the low-risk group from the high-risk group for advanced CRN

Based on the cut-off value, a simplified prediction model for discrimination of the low-risk group from the high-risk group for advanced CRN was constructed (Table 5). The high-risk group had a 3.7-fold increased risk of advanced CRN compared to the low-risk group (1.1% vs. 4.0%, *P* < .001). In the training set, the sensitivity, specificity, accuracy, PPV, and negative predictive value (NPV) of the simplified prediction model were 73.3%, 61.0%, 61.3%, 3.9%, and 99.1%, respectively. In the validation set, the sensitivity, specificity, accuracy, PPV, and NPV were 70.8%, 61.2%, 61.4%, 4.0%, and 98.9%, respectively.

**Table 5:** Discrimination ability of the low-risk group from the high-risk group for advanced colorectal neoplasia.

	Advanced CRN (-), n	Advanced CRN (+), n	*p*	Sensitivity, % (95% CI)	Specificity, % (95% CI)	Accuracy, % (95% CI)	PPV, % (95% CI)	NPV, % (95% CI)
Training set								
Low-risk group, n	11491	107	<.001	73.3 (69.0–77.6)	61.0 (60.3–61.7)	61.3 (60.6–62.0)	3.9 (3.4–4.3)	99.1 (98.9–99.3)
High-risk group, n	7335	294						
Validation set								
Low-risk group, n	11487	124	<.001	70.8 (66.4–75.1)	61.2 (60.5–61.9)	61.4 (60.7–62.1)	4.0 (3.5–4.4)	98.9 (98.7–99.1)
High-risk group, n	7288	300						
Total dataset								
Low-risk group, n	22978	231	<.001	72.0 (68.9–75.1)	61.1 (60.6–61.6)	61.3 (60.9–61.8)	3.9 (3.6–4.2)	99.0 (98.9–99.1)
High-risk group, n	14623	594						

CRN, colorectal neoplasia; PPV, positive predictive value; NPV, negative predictive value.

### Comparison of the discrimination performance of the final model with previous published prediction models for advanced CRN

**Fig 7:**
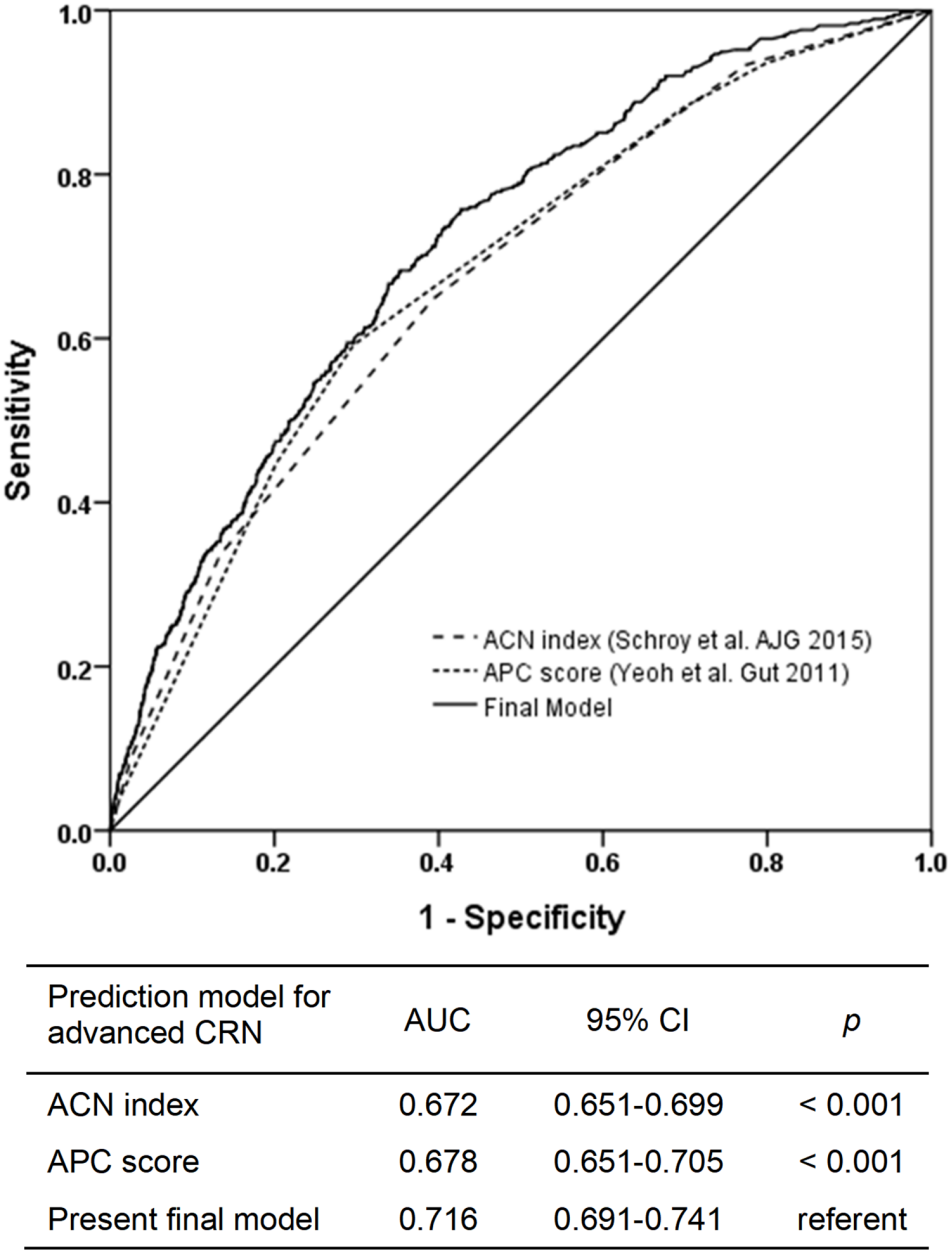
Comparison of the discrimination performance of the final model with previous published prediction models for advanced colorectal neoplasia.

In the validation set, the discrimination performance of the final model was compared with that of the advanced CRN (ACN) index [14] and Asia-Pacific Colorectal Screening score (APCS) [12] using the AUC (Fig 7). The AUC of the final model was 0.716 (95% CI, 0.691–0.741), whereas that of the ACN index was 0.672 (95% CI, 0.645–0.699), and that of the APCS was 0.678 (95% CI, 0.651–0.705). The discrimination performance of the developed model for high-risk patients with advanced CRN was better than that of the ACN index (*P* < .001) or APCS (*P* < .001).

## Discussion

Big data can improve health by providing insights into public health, such as enhanced disease prediction and prevention. Using a big data analytics algorithm, we explored a large health screening examination database. The refined database with structured and unstructured data contained first screening colonoscopy and comprehensive health examination data from 49,450 patients. Big data can not only be applied for verifying alleged associations, but can also be used as a hypothesis-generating machine [24]. In this study, we generated a prediction model for advanced CRN, which might be the first trial for utilization of big data analytics in the field of gastroenterology. The final simplified prediction model was shown to have acceptable discriminative power for patients with advanced CRN. Our simple risk score using easily available information from the patient's clinical questionnaire stratified asymptomatic patients into low- and high-risk groups for advanced CRN before a screening colonoscopy was performed. The discrimination performance of the developed model for high-risk patients with advanced CRN was better than that of existing models.

Based on our results, it is deemed to be inefficient to undergo colonoscopy screening for patients in the low-risk group due to the low probability of advanced CRN as well as the cost and risk associated with colonoscopy. The specificity of our prediction model was not sufficiently high, but the NPVs in this prediction model were as high as 99%. Since this study was populated by asymptomatic individuals who underwent health check-ups, and not symptomatic patients, our objective was to develop and validate a prediction model for estimating the probability of having advanced CRN. We hope to apply this proposed prediction model for the purpose of identifying patients who may not need to undergo a colonoscopy.

There were many studies reporting different risk scoring system for CRC; however, almost none of them can be translated into clinical practice. It is possible because the fecal occult blood test is in fact very convenient, the result is straightforward, and the cost is low. In Korea, a national CRC screening has been in place using fecal immunochemical testing (FIT). The limitation of a stool-based test such as FIT is that it is a diagnostic tool only for the early detection of CRC. Recent guideline grouped the CRC screening tests into cancer prevention and cancer detection tests [25]. The benefits of cancer prevention test can eliminate advanced CRN and prevent CRC. Cancer prevention tests are preferred over detection tests. The goal of CRC screening shifted from “screening detection to prevention by polypectomy [26].” As such, the present study aimed to develop and validate a prediction model for estimating the probability of having advanced CRN and not CRC. Therefore, we think it is difficult to directly compare the predictive model based on FIT with a colonoscopy.

The issue of developing a prediction model for advanced CRN is not novel and several other models already exist. Our study has implemented a predictive model using varied clinical variables acquired in real-world clinical practice. Our prediction model showed more effective prediction for advanced CRN than previous proposed advanced CRN prediction models. We chose to compare our prediction model to studies by Schroy et al [14]. and Yeoh et al [12]. The reason we chose the studies by Schroy et al [14]. and Yeoh et al [12]. is because both studies evaluated advanced CRN predictability and were well designed. The study by Imperidale et al. was also a well-designed study [10], but the outcome measurement was advanced proximal advanced CRN. Therefore, we thought Imperidale’s study was inappropriate for comparison with our model.

Our study was performed with a large population who underwent their first colonoscopy and a comprehensive health screening examination, which may minimize sampling error and represent real-world practice and enhances its usefulness in facilitating shared decision-making for individuals who need CRC screening. The use of EMR systems among healthcare providers has spread widely over the past decade [15]. Using text from EMR system, we applied NLP and the CETAS method to demonstrate the replicability of manual chart review. Previous studies have revealed the utility of NLP in extracting information from clinical text [20–22]. In addition, our risk prediction models use extensive independent variables to estimate the probability of having or developing advanced CRN. Therefore, the discrimination performance of our model for high-risk patients with advanced CRN was better than that of existing models.

Our study had some limitations. External validation could not be performed, so there are concerns about overfitting and generalizability. In addition, the model was developed using a database of patients willing to undergo screening colonoscopy (It is a selected population of 70,959 subjects who underwent colonoscopy screening. It is furthermore selected once more because 21,509 subjects are excluded from the analysis); on that account, it is unclear whether our model can apply to the patients unable or unwilling to undergo colonoscopy. Our study population was quite young for routine screening colonoscopies. The mean age of study population was 50 years old and this may explain why the overall rate of advanced CRN of 2.3% in this study. However, all included subjects underwent colonoscopy as a part of their health check-up. So, even though the patients were young, they did not have symptoms or a family history of CRC. Furthermore, given the long time needed for an adenoma to progress to a carcinoma, the increased number of cases of CRC diagnosed in this age group may originate from adenomas present in individuals in their 40s or earlier [17]. These cancers may be prevented by colonoscopy with polypectomy of premalignant lesions in the preceding decade. Despite this theoretical argument for screening individuals in their 40s or earlier, we included patients who underwent colonoscopies at any age and analyzed the age as continuous variables to develop a prediction model. In addition, we used the mean substitution technique as imputation to deal with missing predictor values in training set. Mean substitution has the benefit of not changing the sample mean for that variable, however mean imputation attenuates any correlations involving the variables that are imputed. The mean imputation has some attractive properties for univariate analysis but becomes problematic for multivariate analysis. Although we used the dataset applied mean substitution technique during univariate logistic regression for the identification of predictors and not used during multivariate logistic regression, the uncertainty in the imputation can lead to overly precise results and errors in our prediction model [27, 28].

Despite these weak points, our model can serve as a clinically useful tool for facilitating shared decision-making related to select the screening modalities for early detection and prevention of CRC, especially when the provider and patient preferences differ. If physicians could predict which patients are at increased risk before colonoscopy, it is possible that they might make better decisions about screening. We developed a simple risk scoring model easily available by questionnaire and precisely identified low- and high-risk groups for advanced CRN at the first screening colonoscopy. This model may increase CRC risk awareness and help healthcare providers encourage the high-risk group to undergo colonoscopy. Furthermore, by identifying the patients with a high risk of advanced CRN, the present model may help to target primary prevention interventions. Once it has been externally validated, the model will be useful to facilitate more effective shared decision-making for CRC screening.
